# Supplementary material for: Deletion of Abi3/Gngt2 influences age-progressive amyloid β and tau pathologies in distinctive ways
Source: Alzheimers Res Ther. 2022 Jul 27;14:104. doi: 10.1186/s13195-022-01044-1 (PMC9327202; doi:10.1186/s13195-022-01044-1)
Supplement: Supplementary file 1 — Additional file 1: Table S1. Description of resources and reagents used in this study. [file 13195_2022_1044_MOESM1_ESM.pdf]

Additional File 1: Table S1: Description of resources and reagents used in this study

REAGENT or RESOURCE	SOURCE	IDENTIFIER
**Antibodies**		
CP27 (total human tau)	Peter Davies	N/A
CP13 (pSer202 tau)	Peter Davies	N/A
MC1 (conformational tau)	Peter Davies	N/A
33.1.1 (Aβ N terminus)	Todd Golde	N/A
Ubiquitin	Dako	Z0458
GFAP (astrocyte)	Dako	Z0334
Iba1 (microglia)	Wako	019-19741
Cd11b (microglia)	Novus	NB11089474
ABI3 antibody	Sigma	HPA017345
S209F ABI3 antibody	Todd Golde (Pacific Immunology)	N/A
FLAG antibody	Sigma	F3165
Synaptophysin	Thermofisher	PA11043
Synaptogyrin 3 (E-11)	Santa Cruz	sc271046
Spinophilin (EIE7R)	Cell Signaling	14136P
PSD95 (D27E11)	Cell Signaling	3450P
GluR2 (L21/32)	Millipore	MABN71
GluR1	Millipore	ABN241
vGlut1	Millipore	MAB5502
CT20	Todd Golde	N/A
GAPDH	Abcam	ab181602
Actin (AC-15)	Sigma	A5441
IRDye secondary antibody<br>Detection: 33.1.1 – HRP (ELISA)	LiCor<br>Todd Golde	925-32212;<br>N/A
Capture:2.1.3 (Aβ42) (ELISA)	Todd Golde	N/A
Capture:13.1.1 (Aβ40) (ELISA)	Todd Golde	N/A
**Bacterial and virus strains**		
AAV1-WT tau	Todd Golde	N/A
AAV1-P301L/S320F tau	Todd Golde	N/A
**Biological samples**		
Mouse brain tissues and lysates	This paper	N/A
**Chemicals, peptides, and recombinant proteins**		
Amyloid β (ELISA standard)	Anaspec	AS-24224, AS-24236
Block Ace	Biorad	BUF 029
ImmPRESS Polymer reagents for IHC	Vector Labs	
Thioflavin S	Sigma-Aldrich	T1892-25G
**Critical Commercial Assays**		
RNAscope 2.5 HDReagent Kit -Red	ACD Biotechne	Cat # 322350
NextSeq 500/550 High Output Kit v2.5 (150 cycles)	Illumina	20024907
TruSeq RNA library preparation kit v2, set A	Illumina	RS-122-2001
DNA 1000 Kit	Agilent	5067-1504
RNA 6000 Nano Kit	Agilent	5067-1511
**Deposited data**		
RNA seq data	This paper	Will be deposited to AD Knowledge Portal (https://adknowledgeportal.org)
**Experimental models: cell lines**		
Not applicable		
**Experimental models: organisms/strains**		
Abi3 +/- mice: B6N(Cg)-Abi3tm1.1(KOMP)Vlcg/J	Jax Labs	Stock #028180
TgCRND8 mice: APP KM670/671NL (Swedish), APP V717F (Indiana)	Tanz Centre for Research in Neurodegenerative Diseases, University of Toronto	N/A
BL/6 mice	Jax Labs	N/A
**Oligonucleotides**		
Abi3 genotyping primer:<br>WT-F 5' - ACC CAG ATC CCT GAG AAT TTG - 3'<br>WT-R 5' - CAA GTC CTG AAG GGA GAA CG- 3'<br>Mu-R 5' - CAG CCC AAG AGG TAG ACA - 3'<br>Mu-F 5' - CGG TCG CTA CCA TTA CCA GT - 3'	IDT	N/A
Mouse Abi3 probe<br>Human Abi3 probe	ACD Bio<br>ACD Bio	Cat No. 539161<br>Cat No. 549711
**Recombinant DNA**		
AAV-human WT tau	Koller et al., 2019	N/A
AAV-human P301L/S320F tau	Koller et al., 2019	N/A

Human ABI3	Origene	RC202853
**Software and algorithms**		
Imagescope (Aperio)	This paper	N/A
STAR v2.6.1a	Dobin et al, 2013	
Rsamtools	Morgan et al, 2018	
GenomicAlignments package	Lawrence et al, 2013	
DESeq2	Love et al, 2014	
WGCNA package in R	Langfelder & Horvath, 2008, 2012	
GeneOverlap package in R	Shen, 2020	
goseq v1.42.0	Young et al, 2010	
