# Supplementary material for: Deletion of Abi3/Gngt2 influences age-progressive amyloid β and tau pathologies in distinctive ways
Source: Alzheimers Res Ther. 2022 Jul 27;14:104. doi: 10.1186/s13195-022-01044-1 (PMC9327202; doi:10.1186/s13195-022-01044-1)
Supplement: Supplementary file 2 — Additional file 2: Table S2. Normalized RNA levels (FKPM values) of Abi3 and Gngt2 from TgCRND8 mice at various ages. [file 13195_2022_1044_MOESM2_ESM.pdf]

Additional File 2: Table S2: Normalized RNA levels of Abi3 and Gngt2 from TgCRND8 mice at various ages

Abi3 (FKPM)	Gngt2 (FPKM)	Mouse_ID	Sex	Age_months	Genotype
0.176381095	0.900590986	240622	F	3	Nontg
0.138958727	0.678966133	363458	F	3	Nontg
0.086048179	0.761551594	366592	F	3	Nontg
0.195148387	0.68692289	366591	F	3	Nontg
0.198230023	0.526318136	357187	F	3	Nontg
0.236406238	0.714431774	366585	M	3	Nontg
0.128697106	0.535333338	366584	M	3	Nontg
0.158314618	0.700565378	406505	F	3	Nontg
0.213704372	0.684797802	357201	M	3	Nontg
0.242039103	0.572891752	357199	M	3	Nontg
0.185533244	0.756195065	357174	M	3	Nontg
0.192791054	0.884340683	357147	M	3	Nontg
0.175545931	0.561034113	363459	F	3	Tg
0.238602512	0.817027617	366597	F	3	Tg
0.137625241	0.742994462	366596	F	3	Tg
0.2181848	0.808081275	366595	F	3	Tg
0.181986321	0.59788622	366581	F	3	Tg
0.207512654	0.738611613	366580	F	3	Tg
0.187774225	0.649828665	368433	M	3	Tg
0.135473115	0.629462801	368432	M	3	Tg
0.182849886	0.485482521	368429	M	3	Tg
0.213087662	0.713252608	368129	M	3	Tg
0.253636496	1.079210246	368125	M	3	Tg
0.159093801	0.977796362	368124	M	3	Tg
0.1422199	0.704265788	169488	M	6	Nontg
0.138733629	0.371581122	NTG_5-1	F	6	Nontg
0.140390115	0.647132114	NTG_5-2	F	6	Nontg
0.183543502	0.596194418	NTG_5-3	F	6	Nontg
0.154453635	0.652412695	NTG_5-4	F	6	Nontg
0.125020043	0.510675698	NTG_5-5	F	6	Nontg
0.208899573	0.658642993	338672	M	6	Nontg
0.144840117	0.679783509	338669	M	6	Nontg
0.104409227	0.577532656	338668	M	6	Nontg
0.123512814	0.499035143	333937	M	6	Nontg
0.169425453	0.446955884	331160	M	6	Nontg
0.171730816	1.072848029	169486	M	6	Nontg
0.293913536	0.741045494	211481	M	6	Tg
0.198031374	1.424016493	209403	M	6	Tg
0.133681899	1.246505616	209404	M	6	Tg
0.249783553	1.38166102	209412	M	6	Tg
0.178933481	1.21245427	168780	F	6	Tg
0.31189088	0.995527295	171320	F	6	Tg
0.22708085	0.772031218	338670	M	6	Tg
0.218509139	1.413212626	331173	F	6	Tg
0.298139809	1.361195267	331171	F	6	Tg
0.188206093	0.867541181	368127	F	6	Tg
0.187983007	0.725204609	366601	M	6	Tg
0.177794087	0.885110493	135969	F	12	Nontg
0.095086004	0.760621783	135970	F	12	Nontg
0.144597971	0.696326077	135990	F	12	Nontg
0.159315147	0.830884448	135994	M	12	Nontg
0.120091504	0.607340001	135995	M	12	Nontg
0.122394468	0.782330545	135996	M	12	Nontg
0.220624225	1.339808433	171973	F	12	Nontg
0.160364504	0.898872807	181718	M	12	Nontg
0.129492449	0.617101757	135980	M	12	Nontg
0.152791243	0.676123635	171974	F	12	Nontg

0.216262742	1.913988636	172622	F	12	Tg
0.37768403	2.433657805	179318	F	12	Tg
0.229540933	0.874675556	181717	M	12	Tg
0.25535607	1.40274375	177181	M	12	Tg
0.158387244	0.813933014	178927	F	12	Tg
0.277987766	1.103504885	328685	M	12	Tg
0.178216086	2.389091943	326639	F	12	Tg
0.354803821	1.869117726	326642	F	12	Tg
0.237257692	1.205440797	326643	F	12	Tg
0.3281244	1.358321219	326644	M	12	Tg
0.219158832	1.892312082	326648	M	12	Tg
0.283550465	1.895767141	326653	F	12	Tg
0.272352912	2.119492362	326657	M	12	Tg
0.390026458	1.862735286	326660	M	12	Tg
0.153351182	0.904801928	253250	F	20	Nontg
0.155414138	0.68773033	253291	F	20	Nontg
0.183572982	1.188043358	256516	F	20	Nontg
0.246918218	2.226069082	253259	F	20	Nontg
0.272109224	0.652233392	253294	M	20	Nontg
0.153150666	1.567213937	140644	F	20	Nontg
0.105520526	1.050623517	140645	F	20	Nontg
0.138030534	1.59340523	140646	F	20	Nontg
0.162859012	0.929903209	184901	M	20	Nontg
0.219005666	0.979666203	184903	F	20	Nontg
0.133643166	0.841594298	184904	F	20	Nontg
0.194590618	1.092924456	184905	F	20	Nontg
0.147889158	1.35560747	184906	F	20	Nontg
0.1068391	1.529578054	184907	M	20	Nontg
0.138284716	0.967244398	184909	M	20	Nontg
0.187190575	0.953852645	184910	M	20	Nontg
0.487008345	1.912256994	253249	F	20	Tg
0.372912178	2.50743311	253290	F	20	Tg
0.290114098	1.96202997	256517	F	20	Tg
0.410572023	2.369793074	253261	F	20	Tg
0.34142956	2.372340115	253254	M	20	Tg
0.316033497	1.540714308	253293	M	20	Tg
0.525720778	2.134806288	256520	F	20	Tg
0.26106499	1.245103673	253276	M	20	Tg

Nontg=nontransgenic; Tg=transgenic
