# Supplementary material for: Deletion of Abi3/Gngt2 influences age-progressive amyloid β and tau pathologies in distinctive ways
Source: Alzheimers Res Ther. 2022 Jul 27;14:104. doi: 10.1186/s13195-022-01044-1 (PMC9327202; doi:10.1186/s13195-022-01044-1)
Supplement: Supplementary file 3 — Additional file 3: Table S3. Normalized RNA levels (FKPM values) of Abi3 and Gngt2 from rTg4510 mice at various ages. [file 13195_2022_1044_MOESM3_ESM.pdf]

Additional File 3: Table S3:Normalized RNA levels of Abi3 and Gngt2 from rTg4510 mice at various ages

Abi3 (FKPM)	Gngt2 (FPKM)	Mouse_ID	Sex	Age_months	Genotype
0.142949629	0.503183108	R716_4	M	2.5	Nontg
0.11015929	0.510683903	R719_6	M	2.5	Nontg
0.08700423	0.469226627	R720_5	M	2.5	Nontg
0.131211413	0.512320348	R819_1	F	2.5	Nontg
0.205599263	0.286420679	R819_5	F	2.5	Nontg
0.128790184	0.498676002	R821_1	F	2.5	Nontg
0.136168394	0.602565091	R710_1	F	2.5	Tg
0.155571449	0.484448246	R814_4	M	2.5	Tg
0.186762708	0.740957274	R816_4	F	2.5	Tg
0.113998286	0.444404463	R817_6	M	2.5	Tg
0.206833575	0.394074097	R819_3	F	2.5	Tg
0.183273882	0.502828544	R821_4	M	2.5	Tg
0.150195425	0.372196585	LP62_4	F	4.5	Nontg
0.113162155	0.470713567	LP8_3	M	4.5	Nontg
0.109831498	0.408699032	LP9_6	M	4.5	Nontg
0.162071572	0.496516468	R706_1	F	4.5	Nontg
0.111103882	0.368738216	R791_3	F	4.5	Nontg
0.143037129	0.548565553	R804_3	M	4.5	Nontg
0.170845651	0.574012933	R706_3	M	4.5	Tg
0.235758543	0.856968577	R708_1	F	4.5	Tg
0.324383648	0.717722585	R796_2	F	4.5	Tg
0.222260613	0.713856612	R798_3	F	4.5	Tg
0.163773122	0.322097804	R803_3	M	4.5	Tg
0.23122546	0.755223872	R804_4	M	4.5	Tg
0.127083655	0.325579037	R697_7	M	6	Nontg
0.162040937	0.51021213	R749_4	M	6	Nontg
0.182842912	0.618728633	R753_2	F	6	Nontg
0.120206902	0.495664984	R760_4	F	6	Nontg
0.208270923	0.63197441	R761_1	F	6	Nontg
0.141346085	0.76763103	R763_5	M	6	Nontg
0.151653016	0.636375426	R_f3_8	M	6	Tg
0.227329158	0.911655627	R_g2_3	F	6	Tg
0.168966609	0.776459768	R695_8	M	6	Tg
0.144749688	0.807078605	R695_9	M	6	Tg
0.22328713	0.874069232	R698_1	F	6	Tg
0.238994993	1.057587858	R773_1	F	6	Tg

Nontg=nontransgenic (Tau-, tTa-); Tg=double transgenic (Tau+, tTa+)
