# Supplementary figures and images for: Deletion of Abi3/Gngt2 influences age-progressive amyloid β and tau pathologies in distinctive ways
Source: Alzheimers Res Ther. 2022 Jul 27;14:104. doi: 10.1186/s13195-022-01044-1 (PMC9327202; doi:10.1186/s13195-022-01044-1)

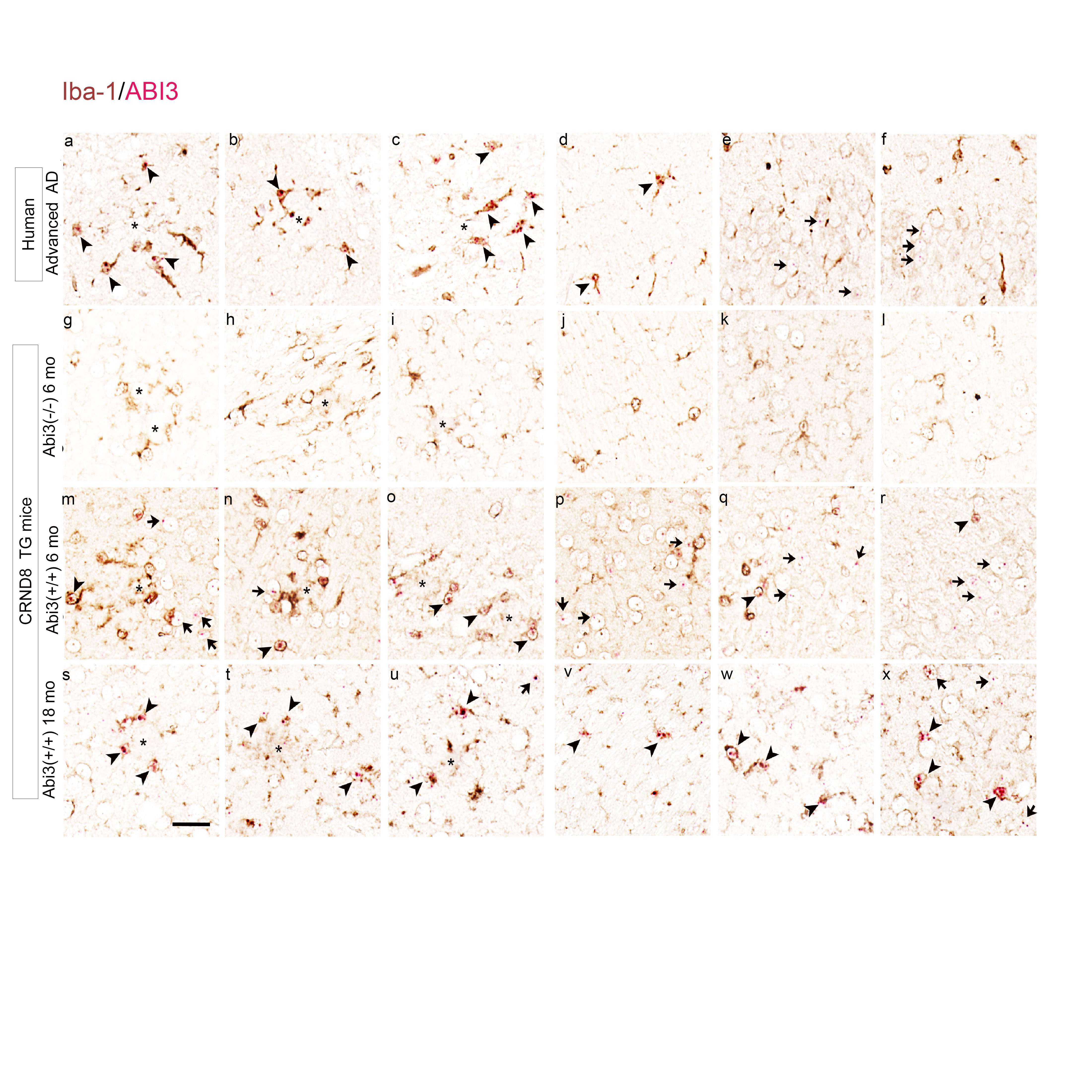

Supplement: Supplementary file 4 — Additional file 4: Fig. S1. Cellular localization of ABI3 RNA in human and mouse. In situ hybridization was done to detect Abi3 RNA on human (a-f) and mouse (g-x) paraffin embedded brain sections. Human or mouse ABI3 specific RNAscope probes were used for in situ hybridization detected by Fast Red (red color) followed by immunohistochemistry for Iba-1 antibody detected by DAB (brown color). Representative forebrain images are shown. Asterisks mark Aβ deposits; arrowheads indicate Iba-1 (microglia) associated in situ signal and arrows indicate in situ signal in non-microglia cells. It should be noted that the presence of amyloid deposits is imputed from the focal clustering of microglia and cellular morphology, in the absence of amyloid staining. n=3 (human AD cases, 6 month old TG-Abi3-Gngt2−/− mice and 6 month old TG-Abi3-Gngt2+/+ mice mice) and n=1 (18 month old TgCRND8 mice, collected independent of this study). Representative of two independent experimental replicates. Please note that this figure also contains the images depicted in Fig 1c-f. TG= transgenic CRND8. Also see Fig. 1. [file 13195_2022_1044_MOESM4_ESM.jpg]

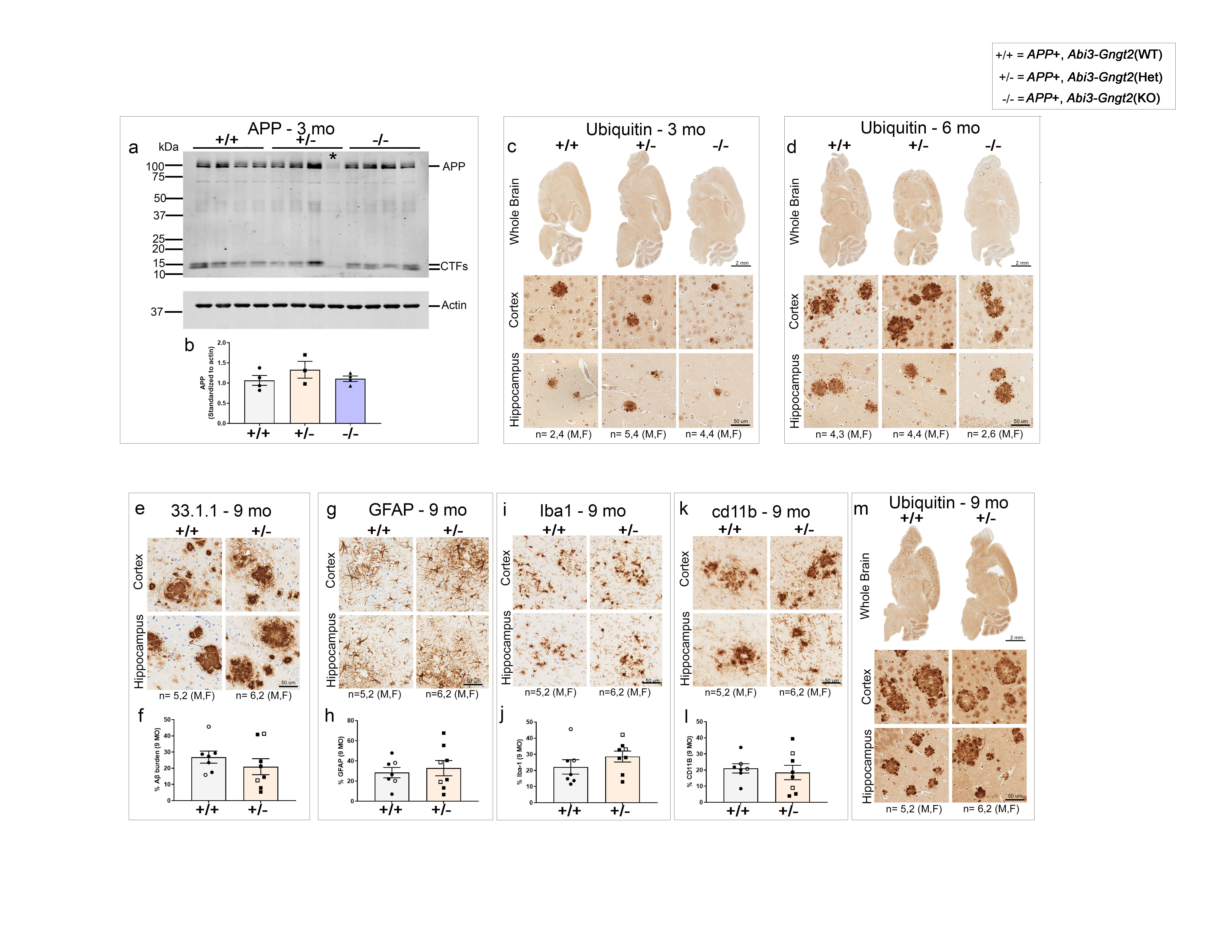

Supplement: Supplementary file 8 — Additional file 8: Fig. S4. Neuropathological attributes of TG-Abi3-Gngt2−/− mice. a-b. Anti CT20 immunoblot indicating full length APP and C terminal fragments (CTF) in APP transgenic mice with WT (+/+), heterozygous (+/−) or KO (−/−) of Abi3-Gngt2 locus (a). Lane marked with asterisk denotes a nonTg mice for APP genotype (a). APP levels normalized to actin is depicted (b). n=3-4 mice/group. Ubiquitin decorating Aβ plaques in 3 month (c) and 6 month old (d) APP TG mice with WT (+/+), heterozygous (+/−) or KO (−/−) of Abi3-Gngt2 locus. Scale Bar, 2mm (whole brain), 50 µm (cortex and hippocampus). e-m. Representative brain images stained with 33.1.1 antibody and corresponding quantitation (e-f), GFAP antibody and corresponding quantitation (g-h), Iba-1 antibody and corresponding quantitation (i-j), cd11b and corresponding quantitation (k-l) and ubiquitin decorated Aβ plaques (m) in 9 month old APP TG mice with WT (+/+) or heterozygous (+/−) Abi3-Gngt2 locus. Scale Bar, 2 mm (whole brain), 50 µm (cortex and hippocampus). N=7-8 mice/genotype. Data represents mean±sem. Clear symbols denote female mice and filled symbols denote male mice. [file 13195_2022_1044_MOESM8_ESM.jpg]

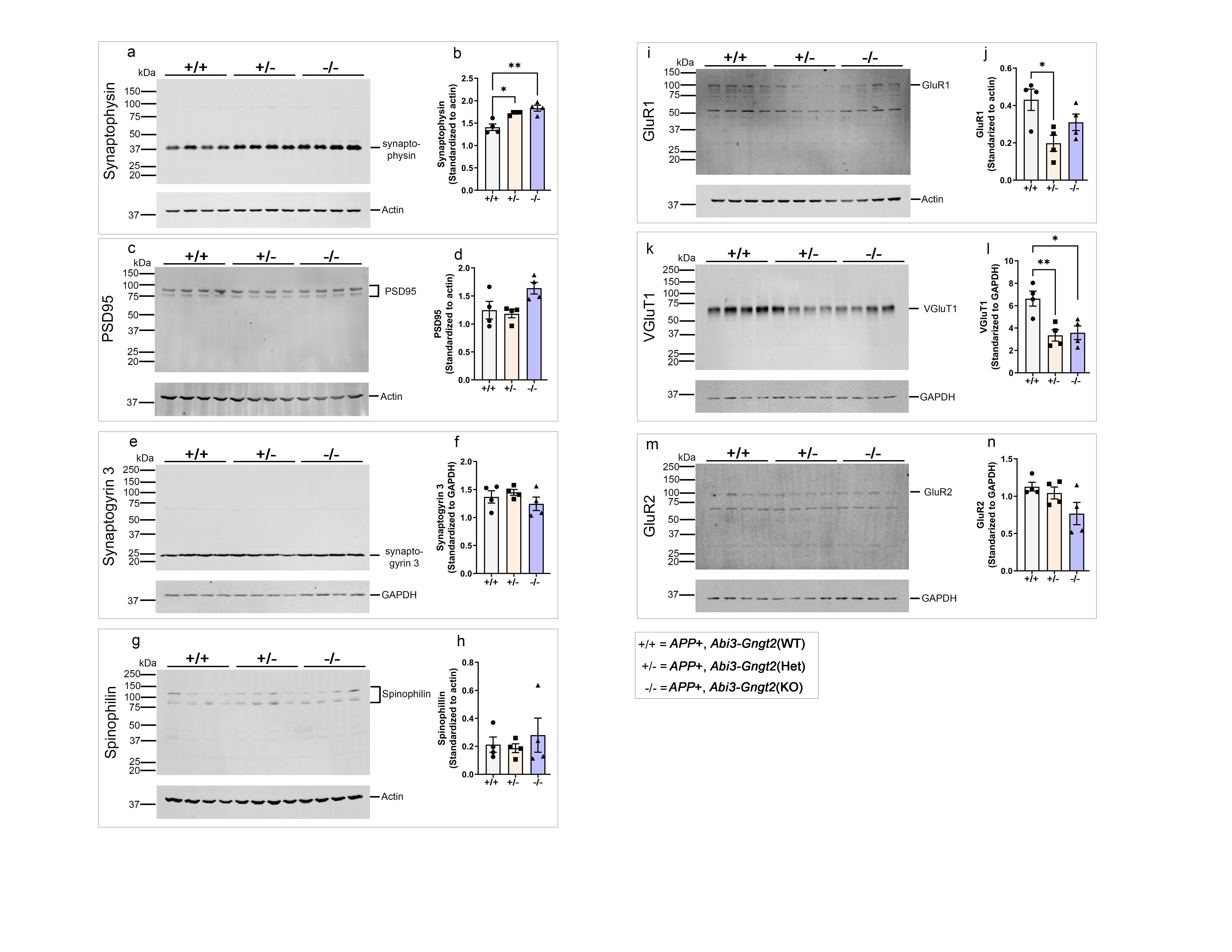

Supplement: Supplementary file 9 — Additional file 9: Fig. S5. Synaptic protein levels in TG-Abi3-Gngt2−/− mice. Immunoblotting depicting levels of synaptic proteins in 3 month old APP Tg mice with WT (+/+), heterozygous (+/−) or KO (-/-) of Abi3-Gngt2 genes (a, c, e, g, i, k, m). Quantitation of the synaptic proteins normalized to actin or GAPDH is depicted (b, d, f, h, j, l, n). Molecular weight markers in kDa are indicated on each panel. N=4 mice/genotype. Data represents mean±sem. 1-way Anova; **p<0.01; *p<0.05. [file 13195_2022_1044_MOESM9_ESM.jpg]

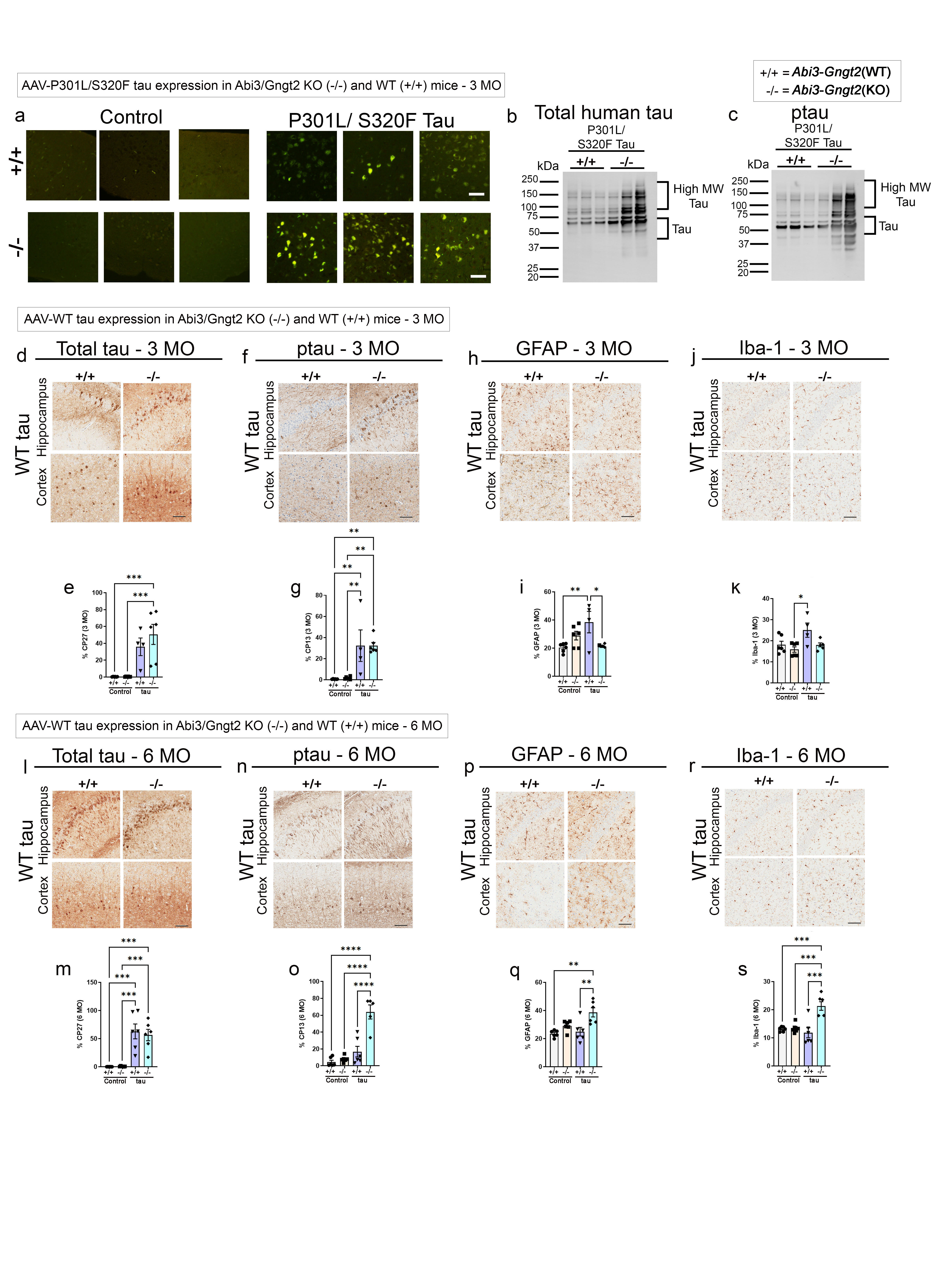

Supplement: Supplementary file 11 — Additional file 11: Fig. S7. Somatic transgenesis modeling of tauopathy in Abi3-Gngt2-/- mice. a-c. Abi3-Gngt2+/+ (WT) and Abi3-Gngt2-/- (KO) mice were injected with control vector (Control) or AAV expressing a double mutant (P301L/S320F) 0N/4R tau in the cerebral ventricles on neonatal day P0 and analyzed at 3 months of age. a. Representative images of ThioS-stained cortex of Abi3-Gngt2+/+ or Abi3-Gngt2-/- mice injected with AAV-P301L/S320F tau or control vector shown. Three individual mice from each cohort are shown. b-c. Guanidine-insoluble brain lysates of AAV-P301L/S320F tau expressing Abi3-Gngt2+/+ or Abi3-Gngt2-/- mice were separated on an immunoblot and probed with CP27 antibody (b) or CP13 antibody (c) to show presence of insoluble NFT tau. N=3 mice/condition. d-s. Abi3-Gngt2+/+ and Abi3-Gngt2-/- mice were injected with control vector (Control) or AAV expressing human WT 0N/4R tau in the cerebral ventricles on neonatal day P0 and analyzed at 3 months (d-k) or 6 months (l-s) of age. Representative brain images of total human tau (detected with CP27 antibody, d, l), ptau (detected with CP13 antibody, f, n), astrocytes (detected with GFAP antibody, h, p) and microglia (detected with Iba-1 antibody, j, r) from cortex and hippocampus of Abi3-Gngt2+/+ or Abi3-Gngt2-/- mice are shown. Quantitative analysis of antibody-stained brain sections (e, g, i, k, m, o, q, s) shown below corresponding stained brain images (d, f, h, j, l, n, p, r). Quantitative data in the graphs depicting the control vector cohort (Abi3-Gngt2+/+ or Abi3-Gngt2-/- genotypes) is shared with corresponding data in Fig. 8 and Fig. 9 as these experiments were done simultaneously. For representative images of the control cohort, please refer to Fig. 8 and Fig. 9. Scale bar, 75 µm. n=4-6 mice/group. Data represents mean±sem. 1-way Anova; ****p<0.0001; ***p<0.001; **p<0.01; *p<0.05. [file 13195_2022_1044_MOESM11_ESM.jpg]
