# Supplementary material for: Deletion of Abi3/Gngt2 influences age-progressive amyloid β and tau pathologies in distinctive ways
Source: Alzheimers Res Ther. 2022 Jul 27;14:104. doi: 10.1186/s13195-022-01044-1 (PMC9327202; doi:10.1186/s13195-022-01044-1)
Supplement: Supplementary file 5 — Additional file 5: Fig. S2. Genomic organization at the Abi3 locus of Mus musculus. a. RNAseq based confirmation of Abi3, Gngt2 and APP levels from three Abi3 genotypes in APP transgenic (APP, Tg) mice or nontransgenic (APP, nTg) mice. Abi3, WT: Abi3+/+; Abi3, het: Abi3+/−; Abi3, hom: Abi3−/−. x axis denotes the genotypes and y axis denotes FPKM values of the corresponding RNA indicated on top of the graph. N=4 mice/genotype. b. The Abi3 knockout mice (Abi3tm1.1(KOMP)Vlcg) was generated by cre-mediated excision of the parental Abi3tm1(KOMP)Vlcg allele resulting in the removal of the neomycin selection cassette, leaving behind the inserted lacZ reporter sequence. The sequence that was excised out on chromosome 11 was located between 95842143 and 95832627 (indicated by black bar). This fragment encompasses the Abi3 coding region and 5’ region of a Gngt2 isoform. [file 13195_2022_1044_MOESM5_ESM.pdf]

a. Transcript copies of Abi3, Gngt2 and human APP in CRND8 mice crossed to Abi3 KO

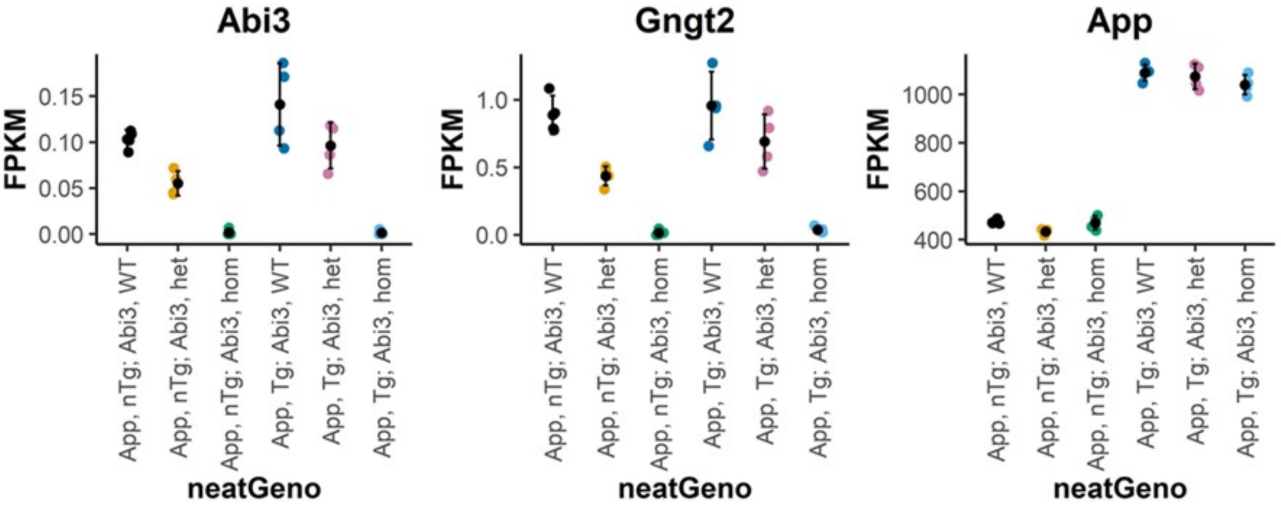

b. Velocigene knockout map of Abi3 KO mice

VelociGene Knockout: velocigene.com/komp/detail/11588

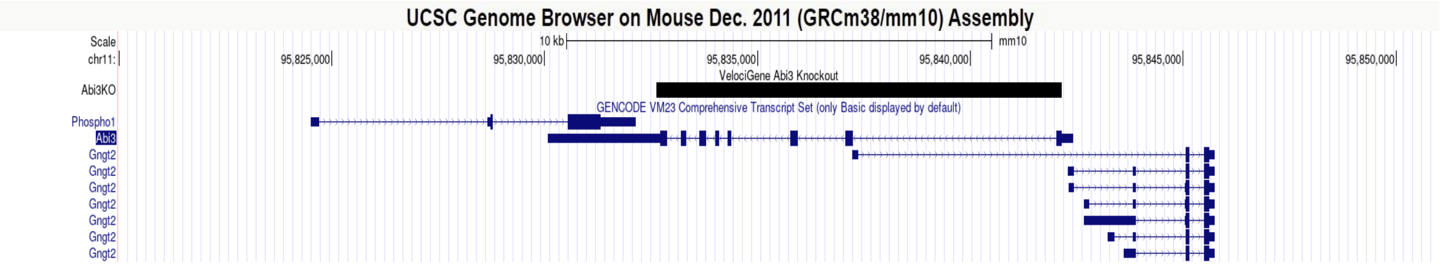

**Gene**	Abi3
**Chromosome**	11
**Deletion Start**	95,842,143
**Deletion End**	95,832,627
**Deletion Size**	9,517
**Genome Build**	38
