# Supplementary material for: Deletion of Abi3/Gngt2 influences age-progressive amyloid β and tau pathologies in distinctive ways
Source: Alzheimers Res Ther. 2022 Jul 27;14:104. doi: 10.1186/s13195-022-01044-1 (PMC9327202; doi:10.1186/s13195-022-01044-1)
Supplement: Supplementary file 6 — Additional file 6: Table S4. Analysis of Abi3 and Gngt2 expression changes in mouse studies (transcriptomic data originally reported by Wan et al. [33] and Al Ouran et al [34] and available at http://mmad.nrihub.org). [file 13195_2022_1044_MOESM6_ESM.pdf]

Additional File 6: Table S4: Analysis of Abi3 and Gngt2 expression changes in mouse studies

StudyID	Abi3	Gngt2	disease	Genotype	Sex	Age	Brain_region	Cell	PMID	study ID
M133	up	up	other	wt synaptoneurosomal fraction; SS	M	na	frontal pole	na		GSE73018
M16	up	up	FTD-ALS	Atg7 cWT; SOD1G93A	na	150d	spinal cord	na	28904095	GSE100888
M181	up	up	other	nmf205-/-;Gcn2-/-	na	3w	cerebellum	na	27085088	GSE79929
M212	up	up	other-neurode	wt untreated	F	P14	na	microglia	28618077	GSE99622
M213	up	up	other-neurode	wt untreated	M	P14	na	microglia	28618077	GSE99622
M214	up	up	other-neurode	wt untreated	M	P4	na	microglia	28618077	GSE99622
M245	up	up	AD	TgCRND8	F	12m	forebrain	na		syn3157182
M246	up	up	AD	TgCRND8	F	20m	forebrain	na		syn3157182
M247	up	up	AD	TgCRND8	F	6m	forebrain	na		syn3157182
M248	up	up	AD	TgCRND8	M	12m	forebrain	na		syn3157182
M45	up	up	other-cell type	Aldh1l1-BAC-eGFP	na	na	cortex	endothelial	25186741	GSE52564
M46	up	up	other-cell type	Aldh1l1-BAC-eGFP	na	na	cortex	myelinating ol	25186741	GSE52564
M47	up	up	other-cell type	Aldh1l1-BAC-eGFP	na	na	cortex	microglia	25186741	GSE52564
M50	up	up	other-cell type	Aldh1l1-BAC-eGFP	na	na	cortex	oligodendrocy	25186741	GSE52564
M60	up	up	other	APP/PS1-21;SAHA-WT;VEH	na	10m	hippocampus	na	26280576	GSE63943
M61	up	up	other	APP/PS1-21;VEH-WT;VEH	na	10m	hippocampus	na	26280576	GSE63943
M65	up	up	AD	CK-6wk-p25	F	3m	hippocampus	na	25693568	GSE65159
M129	down	down	other-cell type	Aldh1l1-GFP subpopulation E	na	na	brain stem	astrocytes	28166219	GSE72826
M136	down	down	other	wt ethanol-ethanol v saline-saline; P2	M	na	frontal pole	na		GSE73018
M138	down	down	other	wt saline-ethanol v saline-saline; P2	M	na	frontal pole	na		GSE73018
M143	down	down	other-inflamm	microglia-wt lps	na	na	whole brain	na	27097852	GSE75246
M15	down	down	other	Atg7 cKO; SOD1G93A /cWT; SOD1G93A	na	150d	spinal cord	na	28904095	GSE100888
M44	down	down	other-cell type	Aldh1l1-BAC-eGFP	na	na	cortex	astrocytes	25186741	GSE52564
M48	down	down	other-cell type	Aldh1l1-BAC-eGFP	na	na	cortex	neurons	25186741	GSE52564
M145	up	down	AD	PS2APP	F	13m	na	astrocytes	27097852	GSE75431
M187	up	down	other-neurode	Q111Q111-Sp1-OE	na	na	striatum	neurons		GSE84055

Website:
http://mmad.nrihub.org
