# Supplementary material for: Deletion of Abi3/Gngt2 influences age-progressive amyloid β and tau pathologies in distinctive ways
Source: Alzheimers Res Ther. 2022 Jul 27;14:104. doi: 10.1186/s13195-022-01044-1 (PMC9327202; doi:10.1186/s13195-022-01044-1)

a. DEG

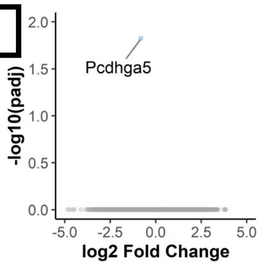


| gene | log2FC | padj |
|---|---|---|
| Pcdhga5 | -0.81 | 1.50E-02 |

b. antiquewhite2

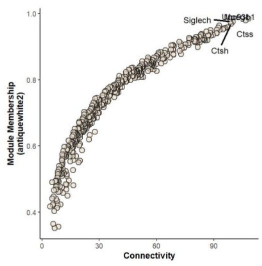


| gene | kWithin | MM | GS_Genotype | GS.GFAP |
|---|---|---|---|---|
| Unc93b1 | 108.33 | 0.9845 | 0.6324 | 0.6213 |
| Ctss | 106.80 | 0.9799 | 0.7585 | 0.7107 |
| Mpeg1 | 100.11 | 0.9754 | 0.7831 | 0.6399 |
| Ctsh | 99.06 | 0.9692 | 0.7264 | 0.6251 |
| Siglech | 98.85 | 0.9756 | 0.6847 | 0.6973 |
| Pld4 | 97.35 | 0.9588 | 0.7091 | 0.5881 |
| Csf3r | 96.12 | 0.9566 | 0.7397 | 0.6688 |
| Ly86 | 95.37 | 0.9549 | 0.7559 | 0.7143 |
| Itgb2 | 94.87 | 0.9521 | 0.6790 | 0.7252 |
| C1qc | 93.12 | 0.9509 | 0.7203 | 0.7145 |

c. coral2

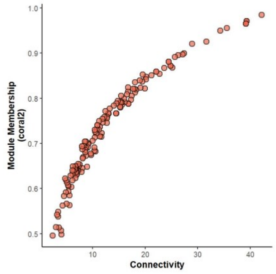


| gene | kWithin | MM | GS_Genotype | GS.GFAP |
|---|---|---|---|---|
| Igkv10-96 | 42.20 | 0.9842 | -0.5495 | -0.6146 |
| Hspd1-ps5 | 39.33 | 0.9703 | -0.5356 | -0.5972 |
| Wfdc21 | 39.33 | 0.9703 | -0.5356 | -0.5972 |
| Gm14240 | 39.33 | 0.9703 | -0.5356 | -0.5972 |
| Gm19967 | 39.33 | 0.9703 | -0.5356 | -0.5972 |
| Olfr1513 | 39.33 | 0.9703 | -0.5356 | -0.5972 |
| Gm31447 | 39.33 | 0.9703 | -0.5356 | -0.5972 |
| Tubal3 | 39.17 | 0.9651 | -0.5452 | -0.6117 |

d. mediumpurple2

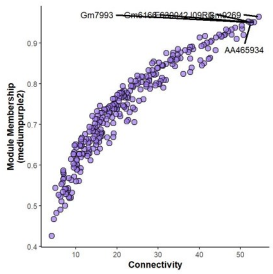


| gene | kWithin | MM | GS_Genotype | GS.Iba1 |
|---|---|---|---|---|
| Gm9269 | 54.53 | 0.9645 | -0.4170 | -0.6351 |
| Gm6166 | 53.31 | 0.9513 | -0.6360 | -0.5025 |
| Gm7993 | 52.64 | 0.9512 | -0.4128 | -0.6585 |
| AA465934 | 52.53 | 0.9493 | -0.6034 | -0.4778 |
| F630042J09Rik | 51.95 | 0.9535 | -0.4760 | -0.6219 |
| Gm6304 | 50.77 | 0.9199 | -0.6993 | -0.4396 |
| Gm15772 | 50.40 | 0.9356 | -0.5744 | -0.5062 |
| Zfp729a | 50.36 | 0.9436 | -0.4126 | -0.7113 |
| Col6a6 | 49.10 | 0.9098 | -0.6086 | -0.5364 |
| Gm5566 | 48.78 | 0.9383 | -0.6122 | -0.5222 |

e. WGCNA and glial profile

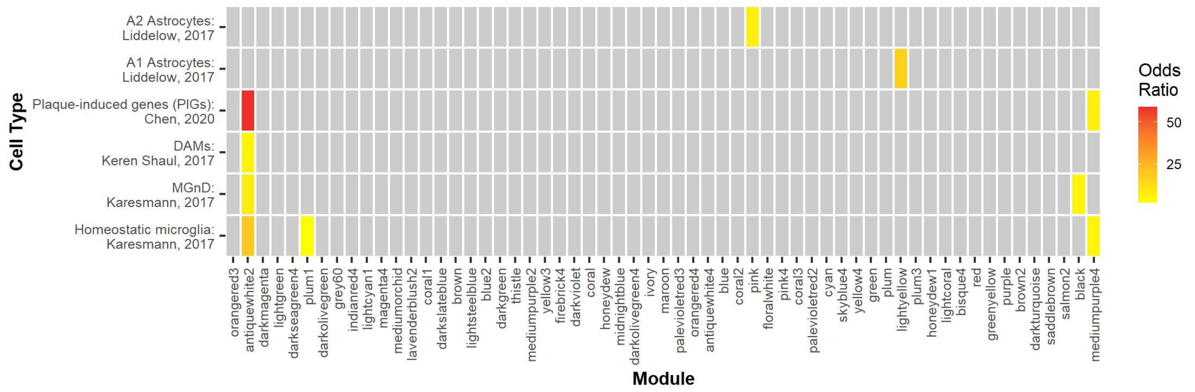


f. KEGG pathways

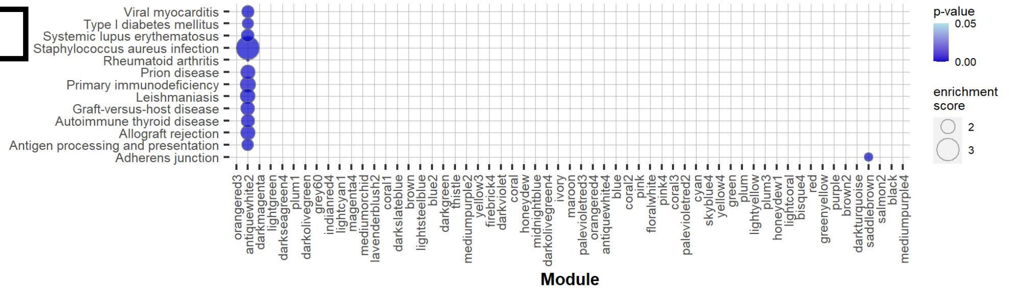

Supplement: Supplementary file 7 — Additional file 7: Fig. S3. Hub genes from WGCNA modules identified in 3 month old Abi3-Gngt2−/− mice. a. Volcano plot and table of altered genes representing DEG in 3 month old heterozygous Abi3-Gngt2+/−vs WT Abi3-Gngt2+/+mice. FC, fold change; padj, adjusted pvalue. b-d. WGCNA module membership is plotted against gene connectivity (kWithin) for genes identified within modules significantly correlated with Abi3-Gngt2 genotype and glial burden traits in Abi3-Gngt2−/−mice. Accompanying table shows top hub genes (as ranked by kWithin values) identified in each co-expression WGCNA modules of 3 month old Abi3-Gngt2−/−mice relative to Abi3-Gngt2+/+ mice. The module members of antiquewhite2, coral2 and mediumpurple2 modules are shown. Module statistics are denoted by: kWithin, extent of gene connectivity within the module; MM, module membership value (of gene to module); GS, gene significance value to specific experimental trait. e. The overlap of genes within WGCNA modules with genes previously identified in AD-associated microglial and astrocytic subtypes is expressed as odds ratio value. The different cell signatures are: neurotoxic A1 and neurotrophic A2 astrocyte [38]; PIG network [21]; DAM [25]; MGnD and homeostatic microglia [24]. Higher odds ratio (warmer color) denotes higher correlation. All p values are adjusted for multiple testing. Grey boxes indicate non-significant odds ratio values. f. Genes within WGCNA modules associated with known KEGG pathways based on over-representation of enriched genes in Abi3-Gngt2−/−mice (relative to Abi3-Gngt2+/+) is depicted in a bubble plot. Pathways with an over-represented p-value ≤ 0.05, the number of module genes within the pathway >5 and an enrichment score >1.5 are depicted. The bubble plot is colored by p-value (blue color; higher p value is indicated by deeper blue hues) and sized by the enrichment score (circle diameter). N=4 mice (2 male, 2 female) per cohort. [file 13195_2022_1044_MOESM7_ESM.pdf]
