# Supplementary material for: Deletion of Abi3/Gngt2 influences age-progressive amyloid β and tau pathologies in distinctive ways
Source: Alzheimers Res Ther. 2022 Jul 27;14:104. doi: 10.1186/s13195-022-01044-1 (PMC9327202; doi:10.1186/s13195-022-01044-1)

a. KEGG pathways

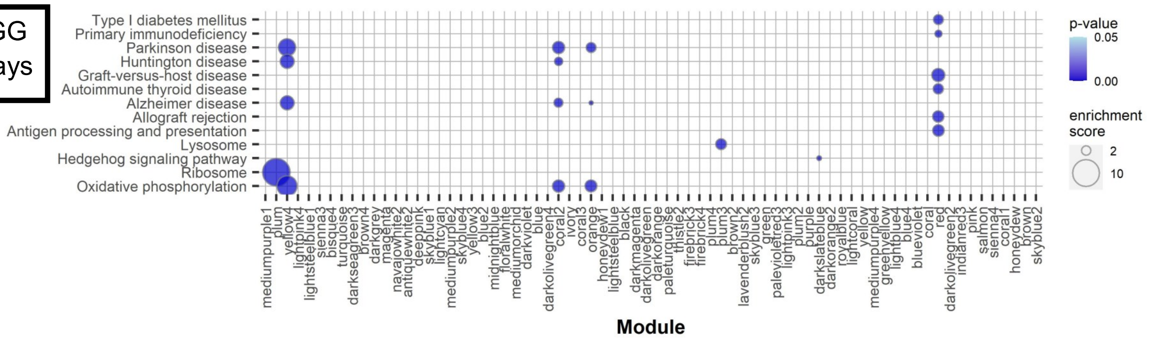

b. honeydew1

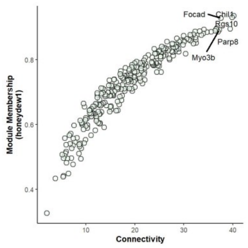

gene	kWithin	MM	GS.Genotype	GS.Ab33.1.1	GS.FA42	GS.SDS42
Chil1	40.23	0.9359	0.5740	-0.6919	-0.5517	-0.5347
Rgs10	39.83	0.9311	0.8073	-0.7290	-0.6468	-0.5994
Parp8	39.44	0.8956	0.5348	-0.4698	-0.6037	-0.5023
Focad	37.57	0.9289	0.6778	-0.8101	-0.5897	-0.5675
Myo3b	37.22	0.8926	0.5865	-0.6043	-0.4158	-0.4585
Zswim6	37.00	0.8863	0.6313	-0.6085	-0.4941	-0.5353
Hax1	36.71	0.8971	0.6728	-0.6427	-0.6906	-0.6198
Gm35281	36.55	0.8942	0.6051	-0.6766	-0.5392	-0.6357
Bms1	36.41	0.8891	0.6030	-0.6488	-0.5670	-0.5924
Gm16433	35.59	0.8860	0.7505	-0.5688	-0.6274	-0.4465

c. plum3

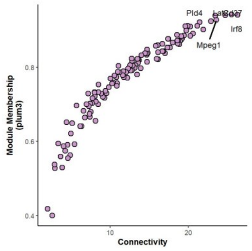

gene	kWithin	MM	GS_Genotype	GS.Ab33.1.1
Irf8	26.31	0.9410	0.6882	-0.8091
Cd37	25.52	0.9418	0.6907	-0.8635
Lat2	24.73	0.9404	0.7682	-0.6935
Mpeg1	23.58	0.9279	0.8503	-0.7983
Pld4	23.43	0.9393	0.7032	-0.7893
Rasal3	22.88	0.9243	0.7065	-0.8359
Traf3ip3	21.91	0.9017	0.6919	-0.7204
Cd300c2	21.42	0.9215	0.6777	-0.6117
Nckap1l	21.14	0.9117	0.7155	-0.8237
AU020206	20.58	0.8907	0.7468	-0.6455

d. sienna3

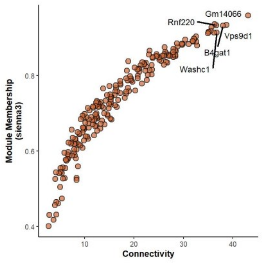

gene	kWithin	MM	GS_Genotype	GS.Ab33.1.1	GS.FA42	GS.SDS40
Gm14066	43.12	0.9587	-0.7463	0.5507	0.5827	0.5398
Vps9d1	38.72	0.9340	-0.6956	0.4789	0.5953	0.5991
B4gat1	37.98	0.9324	-0.7993	0.6457	0.7437	0.7076
Washc1	36.73	0.9136	-0.5923	0.3927	0.4948	0.4898
Rnf220	36.67	0.9334	-0.8766	0.6560	0.5669	0.4833
Il17ra	36.29	0.9353	-0.7078	0.6513	0.5135	0.4034
Tnfsf13os	36.19	0.9133	-0.5398	0.4295	0.5254	0.4776
Wdr90	35.69	0.9274	-0.7712	0.5125	0.6561	0.5825
Zfp13	35.56	0.9184	-0.6325	0.4790	0.5295	0.4496
Gm12958	35.13	0.9153	-0.6790	0.5222	0.7390	0.6439

e. plum4

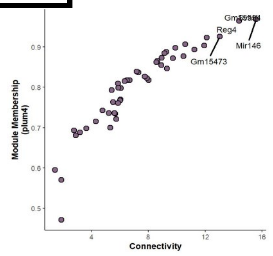

gene	kWithin	MM	GS_Genotype	GS.Iba1
Sval1	15.59	0.9685	0.5081	0.6075
Mir146	15.59	0.9685	0.5081	0.6075
Gm15554	15.59	0.9685	0.5081	0.6075
Reg4	14.39	0.9646	0.5059	0.6283
Gm15473	13.02	0.9259	0.4670	0.6919
Gm14020	12.11	0.9230	0.6467	0.5420
Gm829	11.92	0.9032	0.6712	0.5985
BC024386	11.23	0.8932	0.6639	0.5693
Osr2	11.23	0.8932	0.6639	0.5693
Entpd1	10.59	0.9069	0.3967	0.7157

f. WGCNA and glial profile

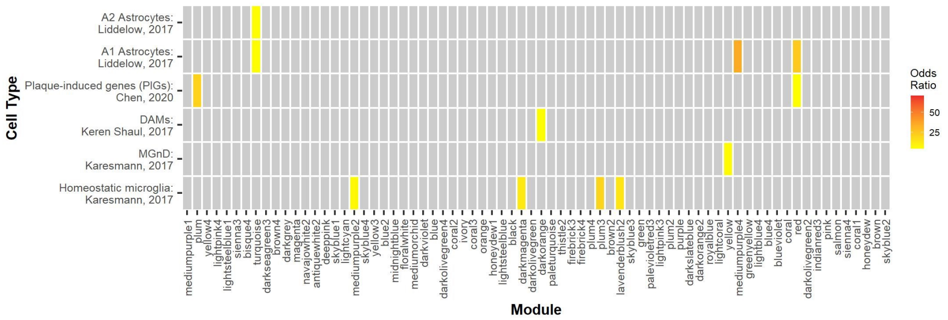

Supplement: Supplementary file 10 — Additional file 10: Fig. S6. Hub genes from WGCNA modules identified in 3 month old TG-Abi3-Gngt2-/- mice. a. Genes within WGCNA modules associated with known KEGG pathways based on over-representation of enriched genes in TG-Abi3-Gngt2-/-mice (relative to TG-Abi3-Gngt2+/+) is depicted in a bubble plot. Pathways with an over-represented p-value ≤ 0.05, the number of module genes within the pathway >5 and an enrichment score >1.5 are depicted. The bubble plot is colored by p-value (blue color; higher p value is indicated by deeper blue hues) and sized by the enrichment score (circle diameter). b-e. WGCNA module membership is plotted against gene connectivity (kWithin) for genes identified within modules significantly correlated with Abi3-Gngt2 genotype and related neuropathology traits in TG-Abi3-Gngt2-/-mice. The top hub genes (as ranked by kWithin values) identified in each co-expression WGCNA modules of 3 month old TG-Abi3-Gngt2-/- mice relative to TG-Abi3-Gngt2+/+ mice. The module members of honeydew1, plum3, sienna3 and plum4 modules are shown, with top hub genes tabulated. Module statistics are denoted by: kWithin, extent of gene connectivity within the module; MM, module membership value (of gene to module); GS, gene significance value to specific experimental trait. f. The overlap of genes within WGCNA modules with genes previously identified in AD-associated microglial and astrocytic sub-types signatures is expressed as odds ratio value. Higher odds ratio is indicated by warmer color. The different cell signatures are: neurotoxic A1 and neurotrophic A2 astrocyte [38]; Plaque-induced gene (PIG) network [21]; Disease-associated microglia (DAM), microglial neurodegenerative phenotype (MGnD) and homeostatic microglia [2425]. All p values are adjusted for multiple testing (padj). Higher odds ratio denotes higher correlation. Grey boxes indicate non-significant odds ratio values. 4 mice (2 male, 2 female) per cohort. [file 13195_2022_1044_MOESM10_ESM.pdf]
